# Zinc Phthalocyanine Photochemistry by Raman Imaging, Fluorescence Spectroscopy and Femtosecond Spectroscopy in Normal and Cancerous Human Colon Tissues and Single Cells

**DOI:** 10.3390/molecules25112688

**Published:** 2020-06-10

**Authors:** Beata Brozek-Pluska, Arkadiusz Jarota, Rafal Kania, Halina Abramczyk

**Affiliations:** Laboratory of Laser Molecular Spectroscopy, Institute of Applied Radiation Chemistry, Lodz University of Technology, Wroblewskiego 15, 93-590 Lodz, Poland; arkadiusz.jarota@p.lodz.pl (A.J.); rafal_kania_1@yahoo.pl (R.K.); halina.abramczyk@p.lodz.pl (H.A.)

**Keywords:** colon cancer, Raman imaging, Raman spectroscopy, photosensitizer, photochemistry

## Abstract

Photodynamic therapy is a clinically approved alternative method for cancer treatment in which a combination of nontoxic drugs known as photosensitizers and oxygen is used. Despite intensive investigations and encouraging results, zinc phthalocyanines (ZnPcs) have not yet been approved as photosensitizers for clinical use. Label-free Raman imaging of nonfixed and unstained normal and cancerous colon human tissues and normal human CCD18-Co and cancerous CaCo-2 cell lines, without and after adding ZnPcS_4_ photosensitizer, was analyzed. The biochemical composition of normal and cancerous colon tissues and colon cells without and after adding ZnPcS_4_ at the subcellular level was determined. Analyzing the fluorescence/Raman signals of ZnPcS_4_, we found that in normal human colon tissue samples, in contrast to cancerous ones, there is a lower affinity to ZnPcS_4_ phthalocyanine. Moreover, a higher concentration in cancerous tissue was concomitant with a blue shift of the maximum peak position specific for the photosensitizer from 691–695 nm to 689 nm. Simultaneously for both types of samples, the signal was observed in the monomer region, confirming the excellent properties of ZnPcS_4_ for photo therapy (PDT). For colon cell experiments with a lower concentration of ZnPcS_4_ photosensitizer, c = 1 × 10^−6^ M, the phthalocyanine was localized in mitochondria/lipid structures; for a higher concentration, c = 9 × 10^−6^ M, localization inside the nucleus was predominant. Based on time-resolved experiments, we found that ZnPcS_4_ in the presence of biological interfaces features longer excited-state lifetime photosensitizers compared to the aqueous solution and bare ZnPcS_4_ film on CaF_2_ substrate, which is beneficial for application in PDT.

## 1. Introduction

Phthalocyanines are important photosensitizers in medical photo diagnostic and photo therapy (PDD, PDT). The unique physical and chemical properties of phthalocyanines, such as their similarity in structure to the biological molecules (chlorophyll, hemoglobin), diversity in chemical structure (central atoms, substituents), near-infrared absorption, high excitation coefficients (typically >10^5^ L mol^−1^cm^−1^), and intrinsic capability to self-assemble, have increased interest in this group of compounds for many years and are crucial for their applications in medicine [1,2,3,4,5,6,7,8,9,10,11,12,13,14,15,16,17,18,19,20].

Every year, the World Health Organization reports thousands of new cases of cancer and around 10 million cancer deaths. Colorectal cancer is the top-ranking cancer, with a ca. 60% mortality rate worldwide. Colectomy, chemotherapy, and radiotherapy are the primary therapeutic methods. However, the chemotherapeutic drug-based treatment of colon cancer is challenging due to the high metastatic potential of cancerous human colon cells and drug toxicity. This is why all new diagnostics and treatment protocols—designed for early-stage cancer detection and the programming of cancer cells’ destruction—are so promising in the fight against this deadly disease.

PDT is an alternative to chemotherapy and chemoradiotherapy. To be brief, PDT involves the combination of light and a photosensitizer. Both factors are harmless in and of themselves, but when combined with oxygen they produce lethal cytotoxic agents, such as singlet oxygen, that destroy cancer cells. It has been proven that the presence of diamagnetic atoms in the phthalocyanine complex, such as zinc (II), aluminum (III), gallium (III), and silicon, results in high triplet quantum yields (Φ_T_ > 0.4) and long triplet lifetimes (τ_T_ > 100 μs) [21]. Both of these parameters, Φ_T_ and τ_T_, effectively influence the efficiency of singlet oxygen generation [21]. Zinc phthalocyanines (ZnPcs) have additional advantageous characteristics such as high chemical and photochemical stability, and excitation at wavelengths greater than 630 nm, resulting in deep tissue penetration, and low dark toxicity [22,23]. The relatively poor solubility of ZnPcs can be overcome by introducing peripheral and non-peripheral substituents in the Pc framework. Another important disadvantage of Pcs, including ZnPcs, is their intrinsic capability to self-assemble, which effectively reduces the capacity for singlet oxygen production. The photochemical activity is exclusively related to the monomer species [24,25]. Aggregates not only decrease the photoactivity of the Pcs, but also limit access to the neoplastic cells, affecting the bioavailability [26]. This is why additional studies to design new soluble compounds are still necessary. A higher affinity of Pcs to the cancer cells can be obtained due to the specific structure of membranes of cells, e.g., a high content of low-density lipoprotein receptors, which enhances the uptake of photosensitizers [27].

One of the most important factors in PDT is selectivity towards diseased tissue, as only those cells that are simultaneously exposed to the photosensitizer, light, and oxygen are subject to the cytotoxic effect. The selectivity of PDT can be obtained in different ways: by preferential uptake of the photosensitizer by the diseased tissue or by the ability to confine the activation of the photosensitizer by restricting the illumination to a specific tissue region. In general, PDT should allow for the selective destruction of cancers, leaving normal tissue intact. 

To date, only a few phthalocyanines have been approved for clinical use or have reached the stage of clinical testing: aluminum phthalocyanine (Photosens®, Russia), against skin, breast, and lung malignancies and cancers of the gastrointestinal tract; and silicon Pc (Pc4®, USA), for the sterilization of blood components against human colon, breast, and ovarian cancers, and gliomas. Zinc phthalocyanine (CGP55847) has undergone clinical trials (phase I/II in Switzerland) against squamous cell carcinomas of the upper aerodigestive tract.

Raman spectroscopy and imaging are well known as sensitive, nondestructive, and highly structured methods that allow for the simultaneous characterization of the biological tissue samples and their interactions with drugs, including photosensitizers, with minimal impact. It has been shown that Raman spectroscopy and imaging have great potential in cancer identification for many human organs (brain [28,29,30,31], breast [32,33,34,35,36,37], esophageal [38,39], stomach [40], salivary gland [41], cervical [42], and colon [43,44,45]) with high sensitivity and specificity. Simultaneously promising results in Pcs characterization, including their aggregation and selective accumulation, crucial for effective PDT, have been obtained by using Raman spectroscopy and imaging [46,47,48,49,50]. It has also been shown that Raman spectroscopy and imaging can determine effective light doses for PDT, which are safe for noncancerous human tissues surrounding tumor masses and also sufficient to destroy cancer cells [51].

Mechanisms of energy dissipation in biological samples, including systems containing photosensitizers, can be determined by femtosecond spectroscopy. It has been shown that the photochemistry mechanisms observed for normal and cancerous human tissues are different and characterized by long-timescale relaxation processes typical of cancer cells [52,53]. The excited-state lifetime of zinc tetrasulphonated phthalocyanine (ZnPcS_4_) in pure water was found to be fast (<80 ps) due to the formation of aggregates. On the contrary, in micelles the electronic relaxation appears to be longer than 1 ns. The elongation of the lifetime in micelles is advantageous for PDT because ZnPcS_4_ has more time to transfer electronic energy from its triplet state to the triplet state of O_2_, which leads to an increase in the quantum yield of singlet oxygen formation [54].

Despite phthalocyanines having been studied for many years by experimental and theoretical methods, some problems remain to be solved, including selective accumulation in biological systems and interactions with cells and tissues.

In this paper, we will focus on the photochemical properties of ZnPcS_4_ and the interactions of ZnPcS_4_ with normal and cancerous human colon cells and tissues.

## 2. Experimental Methods

### 2.1. Phthalocyanine Synthesis

Zinc tetrasulphonated phthalocyanine was synthesized according to Griffiths and co-workers’ method [52]. In this method, dry 4-sulphophthalic anhydride, urea, ammonium chloride, ammonium molybdate, boric acid, and anhydrous aluminum chloride are introduced to sulfolane, slowly heated, and kept for a few hours at a high temperature. The excess of sulfolane is removed and the residue is dissolved in hot water. Finally, a chromatographically pure dye is obtained. 

### 2.2. Sample Preparation

Tissue samples were collected during routine surgery. The nonfixed, fresh samples were used to prepare 16 μm sections. Specimens of the tissue from the tumor mass and the tissue from the safety margin outside of the tumor mass were prepared for Raman analysis by placing the samples on CaF_2_ windows. Adjacent sections were used for the typical histological analysis. All tissue procedures were conducted under a protocol approved by the institutional Bioethical Committee at the Medical University of Lodz, Poland (RNN/323/17/KE/17/10/2017). Written informed consent was obtained from all patients. Details of the sample preparation and the research methodology were given in our previous papers [32,50,51,52]. There are 30 patients in our database.

### 2.3. Cell Culture

CCD-18Co (CRL-1459) cell line was purchased from ATCC (American Type Culture Collection) and cultured using ATCC-formulated Eagle’s Minimum Essential Medium (catalog No. 30-2003). To make the complete growth medium, fetal bovine serum was added to a final concentration of 10%. Every 2–3 days, a new medium was used. The CaCo-2 cell line was also purchased from ATCC and cultured according to the ATCC protocols. To make the complete growth medium base on Eagle’s Minimum Essential Medium, we added a fetal bovine serum to a final concentration of 20%. The medium was renewed once or twice a week. For all results presented in this manuscript, we recorded the Raman spectra and imaging for paraformaldehyde-fixed cells. The procedure for fixed cells was as follows: cells were seeded onto CaF_2_ windows (25 × 1 mm) at a low density of 10^3^ cells/cm^3^. After 24 h incubation on the CaF_2_ slides of up to half of the samples, ZnPcS_4_-H_2_O solution was added for 30 min. After 30 min, the cells were rinsed with phosphate-buffered saline (PBS, Sigma P-5368, pH 7.4 at 25 °C, c = 0.01 M) to remove any residual medium and an excess photosensitizer that did not penetrate inside the cells to be sure that we analyze only the ZnPcS_4_ accumulated inside the cells, fixed in paraformaldehyde (4% buffered formaldehyde) for 10 min, and washed twice with distilled water. The Raman confocal measurements or femtosecond measurements were made immediately after the preparation of the samples.

### 2.4. Raman Spectroscopy and Imaging

All Raman images and spectra reported in this manuscript were recorded using an Alpha 300 RSA+ confocal microscope (WITec, Ulm, Germany) using a 50-μm core diameter fiber, an Ultra-High-Throughput Spectrometer and CCD Camera Andor Newton DU970NUVB- 353 operating in standard mode with 1600 × 200 pixels at −60 °C with full vertical binning. A 532-nm excitation line—the second harmonic of the Nd:YAG laser—was focused on the sample through 40× dry objective (NA of 0.60) for tissue measurements or 40× water dipping objective (NA of 1.0) for cell measurements. The average laser excitation power was 10 mW, with an integration time of 0.5 s for Raman measurements for the high-frequency region, of 1.0 s for the low-frequency region, and of 0.1 s for fluorescence experiments. An edge filter was used to remove the Rayleigh scattered light. A piezoelectric table was used to record Raman images. Spectra were collected at one acquisition per pixel and 1200 lines/mm diffraction grating. The cosmic rays were removed from each Raman spectrum (model: filter size: 2, dynamic factor: 10) and for the smoothing procedure the Savitzky–Golay method was also implemented (model: order: 4, derivative: 0). Data acquisition and processing were performed using WITec Project Plus. All imaging data were analyzed using the Cluster Analysis (CA) method. Briefly, CA allows for grouping a set of objects (vibrational spectra in our studies) that are similar to each other (in vibrational features in our case). CA was performed using WITec Project Plus software with the Centroid model and the k-means algorithm (each cluster was represented by a single mean vector). Normalization of the data was performed using the Origin software normalization model, divided by norm.

### 2.5. Chemical Compounds for Fluorescence Measurements

Fluorescence measurements were performed using the alpha 300 RSA+ confocal Raman microscope (WITec, Ulm, Germany) described above. Hoechst 33342 (catalog No. B2261, Sigma Life Science, USA) and Red Oil-O (catalog No. O0625, Sigma-Aldrich, MO, USA) were used to visualize cells’ substructures without any additional purification.

### 2.6. Femtosecond Spectroscopy

Transient absorption (TA) measurements were performed using a femtosecond laser setup based on a Ti:sapphire oscillator (Tsunami, Spectra-Physics, 82 MHz, 800 nm, pulse duration < 100 fs), pumped by a diode laser (Millennia Pro, Spectra-Physics, 532 nm, 5 W). The femtosecond pulses from the oscillator were used to seed the regenerative amplifier (Spitfire ACE, Spectra-Physics, 1 kHz, output power: 4 W). The output from the amplifier pumped two optical parametric amplifiers (OPA, Topas Prime, Light Conversion). The outputs from OPAs were directly used in pump-probe experiments. The transient absorption signal ΔA was detected by the photodiode (Thorlabs, DET10A) using lock-in detection (Stanford Research, SR830). The pulse duration was determined to be 150 fs in the sample position, as measured by the cross-correlation between the pump and probe pulses in a sample position. The pump and probe pulse energies in TA experiments were set to ~200 nJ and 15 nJ, respectively.

### 2.7. UV-VIS Spectroscopy

Absorption spectra were recorded with a resolution of 0.5 nm using a Perkin Elmer Lambda 750 spectrophotometer.

## 3. Results and Discussion

In this section, we present the Raman spectroscopy and imaging results for cancerous and normal (noncancerous) human colon tissues from the same patient. Before we formulate general conclusions that may be useful in clinical diagnostics and treatment, we provide data for the patient denoted as ZK in our database, to discuss the most important observations regarding the chemical composition of normal and cancerous colon tissues without and after adding ZnPcS_4_ photosensitizer. We present typical Raman spectra based on the thousands of spectra recorded in our measurements. We focus on the vibrational features typical to human colon cells, normal CCD18-Co, and cancerous CaCo-2 without and after adding the ZnPcS_4_ photosensitizer.

### 3.1. Absorption Spectra

**Figure 1:**
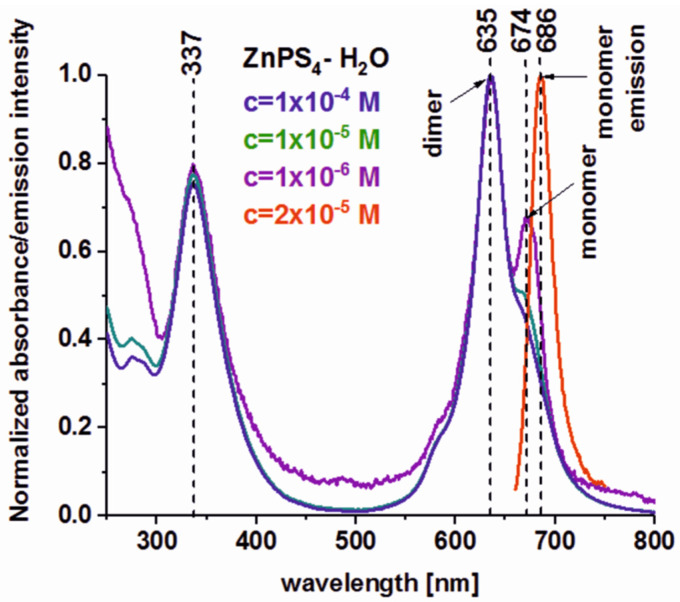
Absorption spectra of aqueous ZnPcS_4_ solutions for the photosensitizer concentrations of c = 1 × 10^−4^, 1 × 10^−5^, and 1 × 10^−6^ M. Fluorescence spectrum of ZnPcS_4_ (c = 2 × 10^−5^ M) is also shown.

Figure 1 shows the absorption spectra of aqueous ZnPcS_4_ solutions for the photosensitizer concentrations of c = 1 × 10^−4^ M (used in our further experiments with human colon tissues), c = 1 × 10^−5^, and 1 × 10^−6^ M (used in our further experiments with human colon cell lines).

The spectra shown in Figure 1 comprise two characteristic strong and broad electronic bands: the Soret band (S_0_→S_2_, π→π*) in the near UV and the Q band (S_0_→S_1_, π→π*) on the red side of the spectrum with maxima at ca. 337, 635, and 674 nm [55].

From a practical point of view and considering the medical applications of phthalocyanines, the most important is an analysis of the Q band, which provides information about the photosensitizer aggregation. One can see from Figure 1 that the Q band has a complex structure. The band at 635 nm should be assigned to the dimer and vibrational progression of monomer, while the band at 674 nm should be assigned to the monomer of ZnPcS_4_. The aggregation of phthalocyanines plays a crucial role in PDT applications because only monomeric forms effectively generate reactive oxygen species (ROS), constituting the basis of PDT [24,25]. One can also see from Figure 1 that, in the concentration range used in our further experiments, the dimerization of ZnPcS_4_–H_2_O, even for the concentration c = 1 × 10^−6^ M, is not negligible. Fortunately, it has been proven that in a human body the dimerization for the aqueous solution of ZnPcS_4_ has definitely shifted in favor of the monomer [49], which promotes the effectiveness of PDT treatment.

**Figure 2:**
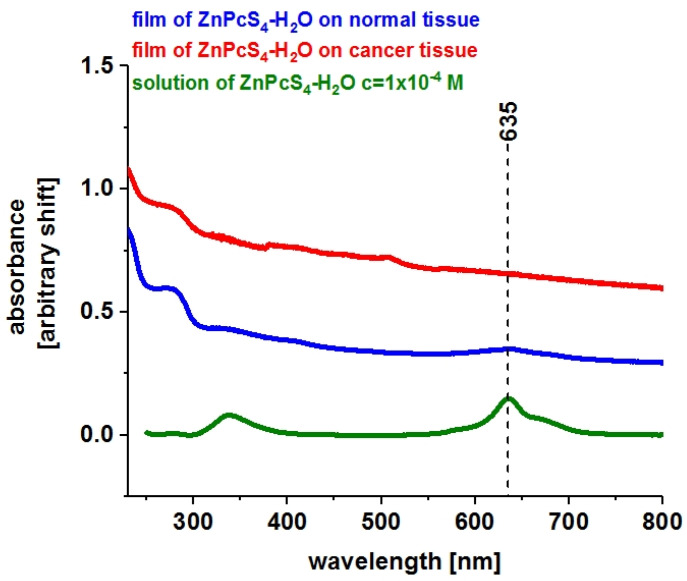
Absorption spectra of ZnPcS_4_ in a form of a thin film produced on normal and cancerous human colon tissue substrates (V = 20 μL, c = 1 × 10^−4^ M) and in aqueous solution (c = 1 × 10^−4^ M).

Figure 2 presents the absorption spectra of aqueous ZnPcS_4_ solution (c = 1 × 10^−4^ M) and ZnPcS_4_–H_2_O in the form of a thin film on human colon tissue substrates from normal and cancerous tissues of the same patient, ZK. 

One can see from Figure 2 that, in contrast to the solutions, the absorption spectra on biological substrates for normal and cancerous human colon tissues are very broad. These spectra are structureless in the Q band region, characteristic of the absorption of monomers, dimers, and higher-order aggregates.

### 3.2. Human Colon Tissue Without ZnPcS_4_

**Figure 3:**
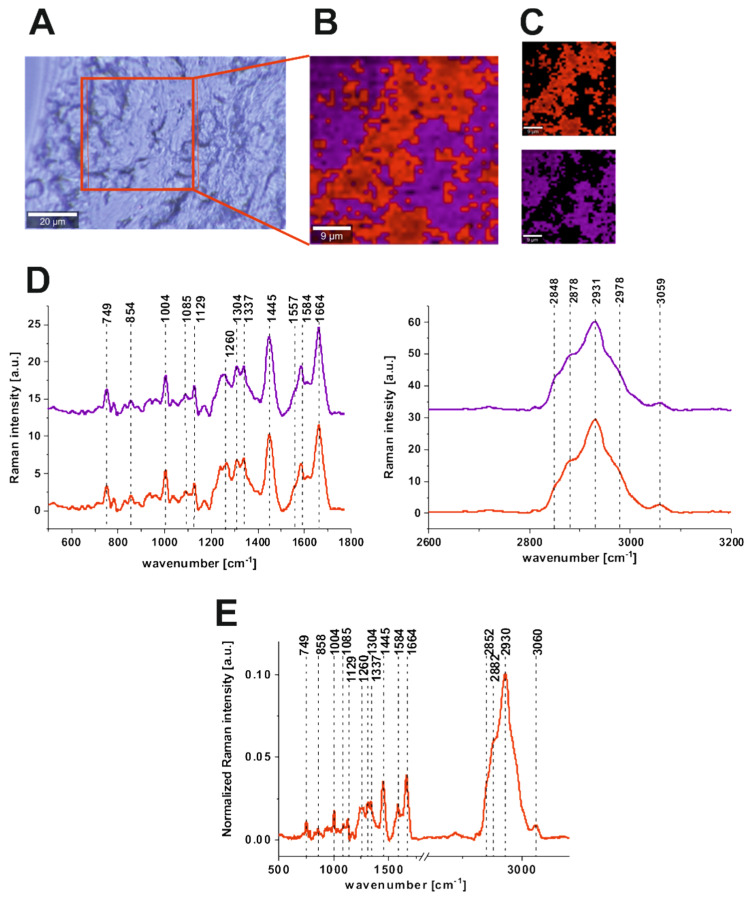
The microscopy image (**A**), Raman image constructed by Cluster Analysis (CA) method (**B**), Raman images of all clusters identified by CA (**C**), average Raman spectra typical of all clusters, colors of the spectra correspond to colors of clusters seen in B and C (**D**), and average (arithmetic mean) Raman spectrum for the entire area of analyzed tissue (**E**) for the cancerous human colon tissue (patient ZK) at 532 nm.

Figure 3 presents the microscopy image, Raman image constructed by CA method, Raman images of all clusters identified by CA, Raman spectra typical of all clusters, and average Raman spectra for cancerous human colon tissue at 532 nm (for no resonant conditions).

**Figure 4:**
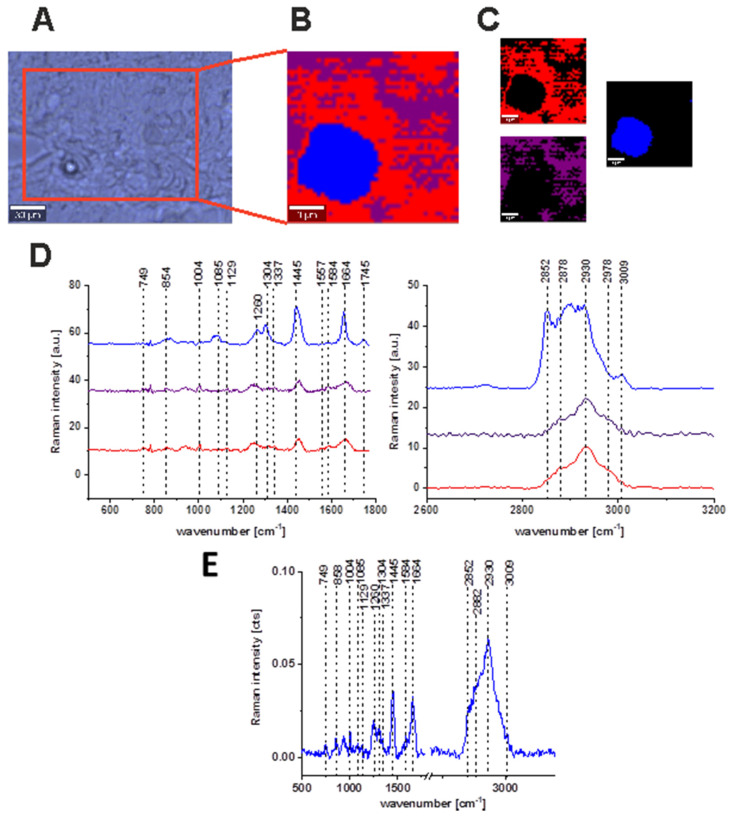
The microscopy image (**A**), Raman image constructed by CA method (**B**), Raman images of all clusters identified by CA (**C**), average Raman spectra typical of all clusters, colors of the spectra correspond to colors of clusters seen in B and C (**D**), and average (arithmetic mean) Raman spectrum for the entire area of analyzed tissue (**E**) for the normal human colon tissue (patient ZK) at 532 nm.

Figure 4 presents the same type of data, obtained by using Raman spectroscopy and imaging for the normal colon tissue from the same patient.

One can see from Figure 3 and Figure 4 that, using Raman spectroscopy and imaging, we can obtain the complex biochemical characteristics of human colon tissue samples based on well-resolved vibrational spectra. It is well known that cancerogenesis affects cellular metabolism. The presented results confirm that Raman spectra can provide relevant information about that reprograming metabolism in a simple way compared to traditional biochemical protocols, because Raman-based techniques are label-free, relatively fast, and objective.

For the cancerous human colon tissue, we used two clusters in CA because the sample was very homogenous; the clusters differ subtly in Raman intensity (see Figure 3). This was the expected result; because the cancerous tissue sample during the surgery was extracted directly from the center of the cancer mass. For the normal human colon tissue, the third cluster, dedicated to the lipid-rich regions of the sample (blue), was added and, as one can see from Figure 4, characterized by totally different vibrational profiles in the low- and high-frequency regions.

The fingerprint (low-frequency) region of Raman spectra shown in panel D of Figure 3 and Figure 4 provides a lot of information on the chemical composition of biological samples because each Raman peak corresponds to specific functional groups of the tissues’ chemical constituents.

The peak at 749 cm^−1^ is typically associated with nucleic acids, DNA, tryptophan, and nucleoproteins [56]; the broad peak with two maxima, ca. 829 and 849 cm^−1^, should be assigned to tyrosine (the Fermi resonance between the first overtone of the aromatic out of plane ring bend and the aromatic breathing fundamental) [57]; the peak at 858 cm^−1^ is typical of the stretching mode of the phosphate group from phosphorylated tyrosine (partially overlapping with the phosphate group from DNA) [57]; 872 cm^−1^ and 938 cm^−1^ are most probably due to single bond stretching vibrations for the amino acids proline and valine and polysaccharides collagen and tryptophan [56]; the sharp peak at 1004 cm^−1^ is associated with phenylalanine [57]; the peak at 1085 cm^−1^ is typical of phosphodiester groups in nucleic acids [57,58]; the signal at 1129 cm^−1^ is characteristic of saturated fatty acids; the band at 1337 cm^−1^ is typical of CH_3_CH_2_ wagging vibrations of collagen [59]; the band at 1445 cm^−1^ is typical of lipids and proteins; the peak at 1585 cm^−1^ is typical of CN_2_ scissoring and NH_2_ rock of mitochondria and phosphorylated proteins [57]; and the peak at 1745 cm^−1^ characterizes the C=O group of lipids and lipids esters. The other, high-intensity group of vibrations can be assigned to lipids, fatty acids, and proteins. Moreover, Raman spectra are secondary structure sensitive [41]. Proteins can be characterized using Raman spectra by peaks typical of Amide I (C=O stretch near 1655 cm^−1^), Amide II (N–H bend + C–N stretch near 1557 cm^−1^, very weak), and Amide III (C–N stretch + N–H bend near 1260 cm^−1^) [57,60]. The recorded high-frequency signals originate in the symmetric and antisymmetric stretching vibrations of C-H bonds of lipids, glycogen, proteins, RNA, and DNA. Lipids and fatty acids, including unsaturated fraction, appear at 2848, 2875, 2882, and 3009 cm^−1^ [32,56,57,60]. The contribution of proteins, in the high-frequency region, is seen specifically at 2875, 2882, and 2930 cm^−1^ [32,56,57,60]. 

Table 1 summarize all our observations.

**Figure 5:**
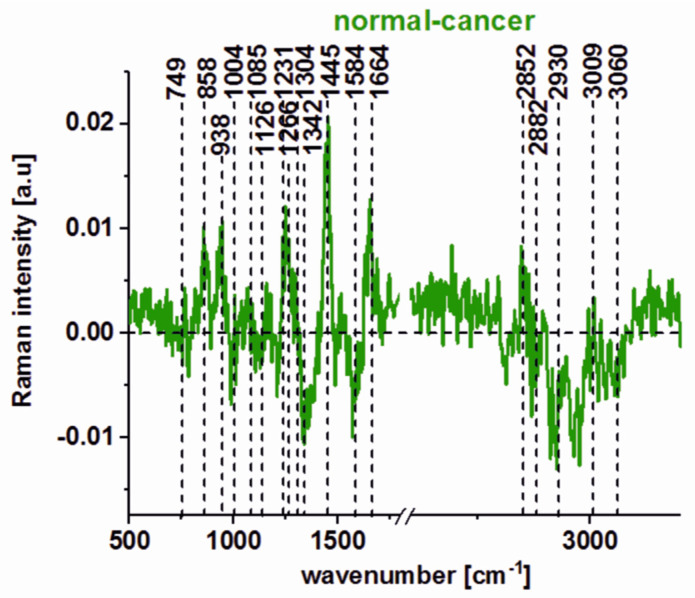
The difference spectrum calculated for normal and cancerous human colon tissues (average spectrum typical of normal tissue minus average spectrum typical of cancerous tissue, based on 4050 Raman single spectra).

To summarize, one can see from Figure 3 and Figure 4 that many peaks are observed for both types of human colon tissues, but this does not mean that the biochemical composition of normal and cancerous human colon tissues is the same. Many qualitative and quantitative differences can be found. The difference spectrum (average spectrum typical of normal tissue minus average spectrum typical of cancerous tissue) is presented in Figure 5. One can see from Figure 5 that the biggest differences can be found for DNA/RNA, phenylalanine, collagen, proteins including phosphorylated proteins, and lipids. The positive peaks in Figure 5 confirm the higher content of DNA/RNA, phenylalanine, collagen, proteins, and phosphorylated proteins in cancerous human colon tissue and the higher content of lipids including unsaturated fraction in the normal one (negative peaks).

### 3.3. Human Colon Tissue After Adding ZnPcS_4_

**Figure 6:**
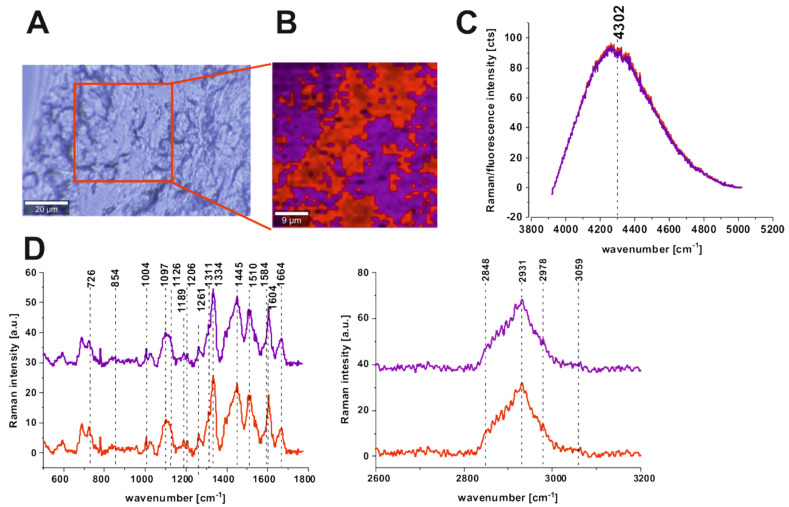
Microscopy image (**A**), Raman image constructed by CA method (**B**), Raman/fluorescence spectra in the range typical of ZnPcS_4_ phthalocyanine fluorescence (**C**) and average Raman spectra for all clusters, for low- and high-frequency ranges for cancerous human colon tissue after adding ZnPcS_4_ phthalocyanine (patient ZK) at 532 nm (**D**).

The same analysis was performed for human colon tissue samples after adding the ZnPcS_4_ photosensitizer (the concentration of the aqueous solution was 1 × 10^−4^ M, the volume used was 20 μL, and the analyzed area was the same as for the tissue without the photosensitizer). Figure 6 presents the microscopy image, Raman image constructed by CA method, and average Raman spectra typical for all clusters for cancerous human colon tissue in the low- and high-frequency ranges and in the range typical of ZnPcS_4_ fluorescence.

One can see from Figure 6 (panel D) that, after adding ZnPcS_4_, using Raman spectroscopy and imaging is still possible to obtain complex information about the chemical composition of human colon cancer tissue. Moreover, together with the Raman spectra typical of human cancer tissue, the fluorescence spectra of ZnPcS_4_ can be recorded (Figure 6C) to give information on the localization, concentration, and aggregation of the photosensitizer and its interactions with constituents of the tissue. As the π-stacked aggregates do not show fluorescence since the relevant electronic transition is forbidden [61], the observed signal arises from ZnPcS_4_ monomers or non-cofacial aggregates.

Similar to the sample without ZnPcS_4_, the spectra for the two clusters are very much alike, which once again confirms the homogeneity of the pathological tissue, removed directly from the tumor mass. From Figure 6C, one can see that the concentration of the ZnPcS_4_ photosensitizer is also very similar in both clusters as the fluorescence intensity is comparable. The fitting procedure confirmed that the maximum of the fluorescence band of ZnPcS_4_ photosensitizer is observed for 4302 cm^−1^ (689 nm for 532 nm excitation wavelength).

**Figure 7:**
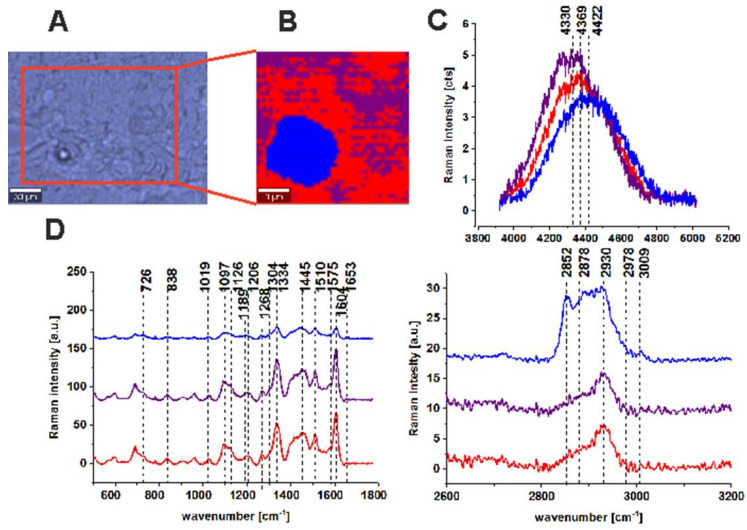
Microscopy image (**A**), Raman image constructed by CA method (**B**), Raman/fluorescence spectra in the range typical of ZnPcS_4_ phthalocyanine fluorescence (**C**) and average Raman spectra for all clusters, for low- and high-frequency ranges for normal human colon tissue after adding ZnPcS_4_ phthalocyanine (patient ZK) at 532 nm (**D**).

Figure 7 presents the results obtained for normal colon tissue.

One can see from Figure 6 and Figure 7 that, for the normal tissue sample, the Raman signal typical of ZnPcS_4_ fluorescence is lower than for cancerous tissue and differentiated in terms of the maximum band position (Figure 6C and Figure 7C). The maximum peak positions typical of phthalocyanine fluorescence observed for the normal colon tissue are 4330 cm^−1^ (691 nm for 532 nm excitation wavelength), 4369 cm^−1^ (693 nm for 532 nm excitation wavelength), and 4422 cm^−1^ (695 nm for 532 nm excitation wavelength). This confirms that, for normal human colon samples, the red shift of the maximum peak position compared to the cancerous tissue (689 nm for 532 nm excitation wavelength) is observed. Moreover, this effect is accompanied by a decrease in the fluorescence intensity, which suggests a lower concentration of the photosensitizer. The lowest concentration for normal human colon tissue was observed for the lipid-rich region. Moreover, adding ZnPcS_4_ phthalocyanine does not disrupt the Raman peaks of chemical constituents typical of normal human colon tissue (Figure 7D).

To summarize, for normal human colon tissue samples in contrast to cancerous ones, we observed a lower affinity to ZnPcS_4_ phthalocyanine (lower Raman/fluorescence signals typical of ZnPcS_4_). The higher concentration in cancerous tissue was concomitant with the blue-shift of the maximum peak position of the photosensitizer (689 nm for cancerous tissue and 691–695 nm for normal tissue), but, simultaneously for both types of samples, the signal was observed in the monomer region, confirming the excellent properties of ZnPcS_4_ for PDT [39,62].

### 3.4. Human Colon Cells Without ZnPcS_4_

**Figure 8:**
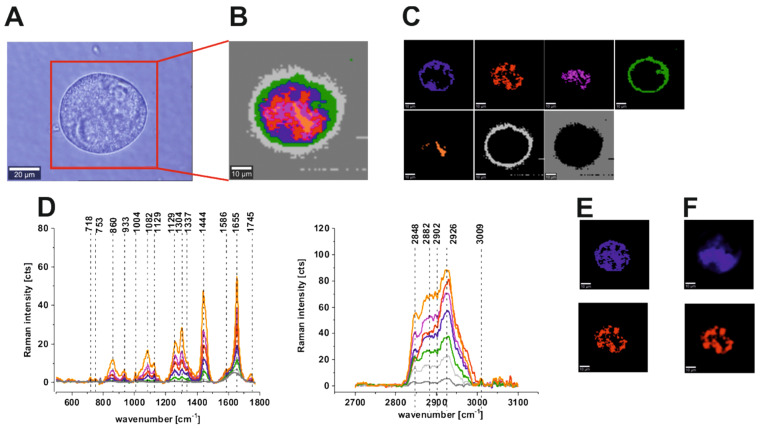
Microscopy image (**A**), Raman image constructed based on CA method (**B**), Raman images of all clusters identified by CA, nucleus (red), mitochondria (magenta), lipid-rich regions (blue and orange), membrane (light gray), cytoplasm (green), cell environment (dark gray) (**C**), Raman spectra typical of all clusters for the low- and high-frequency regions (**D**), Raman clusters typical of all lipid-rich structures (blue), nucleus (red) (**E**) and fluorescence staining (**F**) obtained using Red Oil-O to visualize lipid-rich regions, including lipid droplets (blue) and Hoechst 33342 to visualize nucleus (red) for CaCo-2 human colon cancer cells. The colors of the spectra correspond to the colors of the clusters.

An analogous analysis was made for single cells of the human colon. We performed CA to visualize and analyze vibrational features for all substructures of human cells: the nucleus (red), mitochondria (magenta), lipid-rich regions (blue and orange), membrane (light gray), and cytoplasm (green) in low- and high-frequency regions. Figure 8 presents results obtained for cancer human colon cell line CaCo-2: the microscopy image, Raman image constructed based on CA method, Raman images of all clusters identified by CA, Raman spectra typical of all clusters for the low-frequency and high-frequency regions, Raman clusters typical of lipid-rich structures, and nucleus and fluorescence imaging after cell staining using Hoechst 33342 to visualize the nucleus and Red Oil-O to visualize lipid-rich regions (including lipid droplets) for CaCo-2 human cancer cells; the colors of the spectra correspond to the colors of the clusters.

To compare the results for normal and cancerous cells, we performed the same analysis for CCD-18Co normal human colon cells.

**Figure 9:**
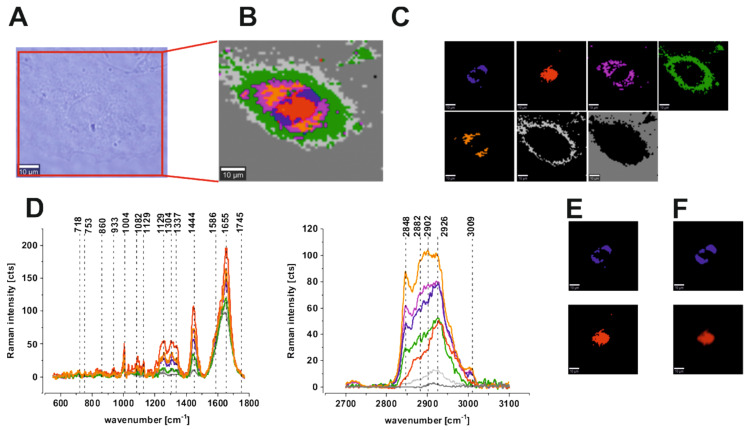
Microscopy image (**A**), Raman image constructed based on CA method (**B**), Raman images of all clusters identified by CA, nucleus (red), mitochondria (magenta), lipid-rich regions (blue and orange), membrane (light gray), cytoplasm (green), cell environment (dark gray) (**C**), Raman spectra typical of all clusters for low- and high-frequency regions (**D**), Raman clusters typical of all lipid-rich structures (blue), nucleus (red) (**E**), and fluorescence staining (**F**) obtained using Red Oil-O to visualize lipid-rich regions including lipid droplets (blue) and Hoechst 33342 to visualize nucleus (red) for CCD18-Co normal human colon cells. The colors of the spectra correspond to the colors of the clusters.

Figure 9 presents the microscopy image, Raman image constructed based on CA method, Raman images of all clusters identified by CA, Raman spectra typical of all clusters for low- and high-frequency regions, Raman clusters typical of lipid-rich structures and nucleus, and fluorescence staining obtained using Hoechst 33342 to visualize the nucleus and Red Oil-O to visualize lipid-rich regions, including lipid droplets for CCD18-Co normal human colon cells. The colors of the spectra correspond to the colors of the clusters.

**Figure 10:**
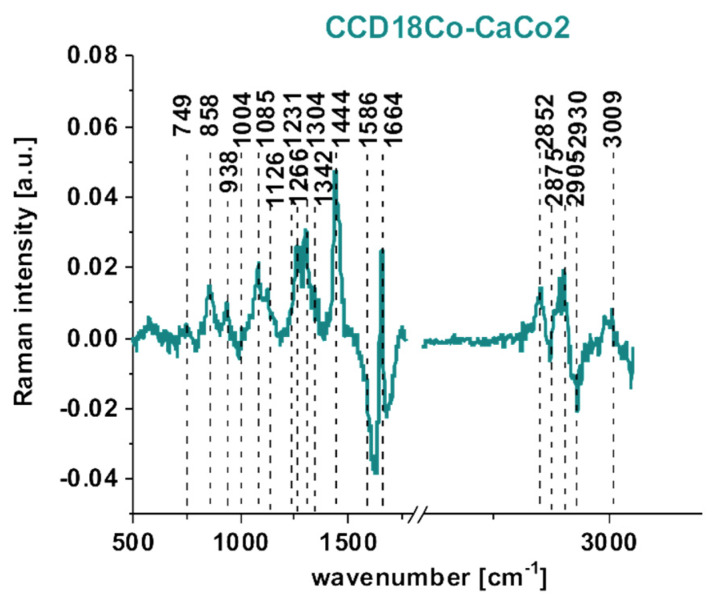
The difference spectrum calculated for normal human colon CCD18-Co and cancerous CaCo-2 cell lines.

One can see from Figure 8 and Figure 9 that Raman imaging provides well-resolved Raman spectra of single colon cells, enabling the characterization of many cell organelles such as the nucleus, mitochondria, lipid-rich regions including lipid droplets, membrane, and cytoplasm. Moreover, Figure 8F and Figure 9F confirm that fluorescence imaging accurately corresponds to the Raman imaging of the nucleus and lipid-rich regions for both normal and cancerous human colon cells, which helps with spectroscopic data interpretation and the correct assignment of different clusters to organelles. For the average spectra of normal and cancer cells, we calculated the difference spectrum, which is presented in Figure 10.

The Amide III (1230–1300 cm^−1^) and Amide I bands (1600–1690 cm^−1^) are widely used to study the secondary structure of proteins and the’ global amount of protein [29,39]. One can see from Figure 10 that these peaks are positive on the difference spectrum, which confirms the higher contribution of proteins for the cancerous CaCo-2 cell line; the other protein-related bands observed at 1004, 1585, and 2926 cm^−1^ also are more intense in the cancerous cells. The higher content of proteins in the cancerous CaCo-2 cell line can be explained by the fact that cancer cells typically have higher RNA/DNA content and an increasing number of studies demonstrate, e.g., the potential use of cell-free DNA as a surrogate marker for multiple indications in cancer, including diagnosis, prognosis, and monitoring [63,64]. The same trend can be observed for the band 858 cm^−1^, assigned to hydroxyproline [57]. All bands associated with the phosphates, ca. 749, 1085, 1586 cm^−1^, also show a higher contribution to the cancer CaCo-2 cell line. The higher phosphorylation status of cancerous tissues has been shown in the literature for many organs including the breast, brain, and colon [57]. In contrast, a negative correlation for the cancerous CaCo-2 colon line can be observed in Figure 10 for lipid peaks from the high-frequency region (2845 cm^−1^), which confirms the reprogramming of lipids’ metabolism in cancer cells [65].

To summarize, chemical differences were found between normal CCD18-Co and cancerous CaCo-2 human colon lines, based on vibrational features, for several chemical constituents: DNA/RNA, lipids, and proteins. The Raman imaging results confirm that vibrational spectra can be used to visualize many organelles of single cells, including the nucleus, cell membrane, lipid structures, mitochondria, and cytoplasm, based on the Cluster Analysis method. Moreover, we show that the Raman imaging results accurately correspond to the fluorescence imaging data, confirming the possibility of analyzing the cells’ substructures without adding external dyes.

### 3.5. Human Colon Cells After Adding ZnPcS_4_

Raman spectroscopy and imaging were also used to investigate the localization and photochemistry of ZnPcS_4_ phthalocyanine in normal and cancerous human colon single cells.

**Figure 11:**
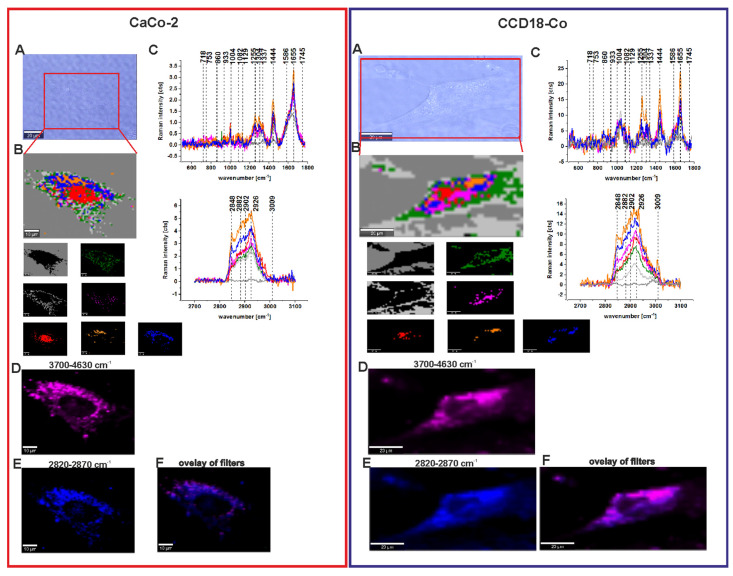
Microscopy image (**A**), Raman image constructed based on CA method and Raman images of all clusters identified by CA, nucleus (red), mitochondria (magenta), lipid-rich regions (blue and orange), membrane (light gray), cytoplasm (green), cell environment (dark gray) (**B**), Raman spectra typical of all clusters for low- and high-frequency regions (**C**), Raman image of ZnPcS_4_ phthalocyanine distribution based on filter 3700–4630 cm^−1^ (**D**), Raman image of the distribution of lipids structures and mitochondria based on filter 2820–2870 cm^−1^, (**E**) overlay of ZnPcS_4_ phthalocyanine, and mitochondria/lipids distribution filters, (**F**) the concentration of ZnPcS_4_ in a medium c = 1 × 10^−6^ M.

Figure 11 presents the Raman imaging data obtained after adding ZnPcS_4_ phthalocyanine for a final concentration in cells of c = 1 × 10^−6^ M for the CCD-18Co and CaCo-2 human colon cell lines.

One can see from Figure 11 that, for the cell lines, after adding the photosensitizer it is still possible to characterize the vibrational features of each cell substructure such as the nucleus, lipid structures, mitochondria, membrane, and cytoplasm (panel B). Moreover, based on the Raman spectra (panel C), it is possible to identify DNA/RNA, proteins, phosphorylated proteins, lipids, and unsaturated lipid signals. However, in this experiment, we mostly focused on the localization of the phthalocyanine inside each cell. One can see from Figure 11 that, based on the Raman imaging data and filters typical of mitochondria and lipids (2820–2870 cm^−1^) in panel E and the Raman/fluorescence signals of ZnPcS_4_ (3700–4630 cm^−1^) in panel D, a perfect match is observed between the localization of mitochondria, lipids structures, and phthalocyanine inside cells. Moreover, this observation is true for both the normal CCD18-Co and cancerous CaCo2-Co human colon cells. This finding is confirmed by Figure 11F, which shows the overlay of mitochondria/lipids and phthalocyanine filters.

**Figure 12:**
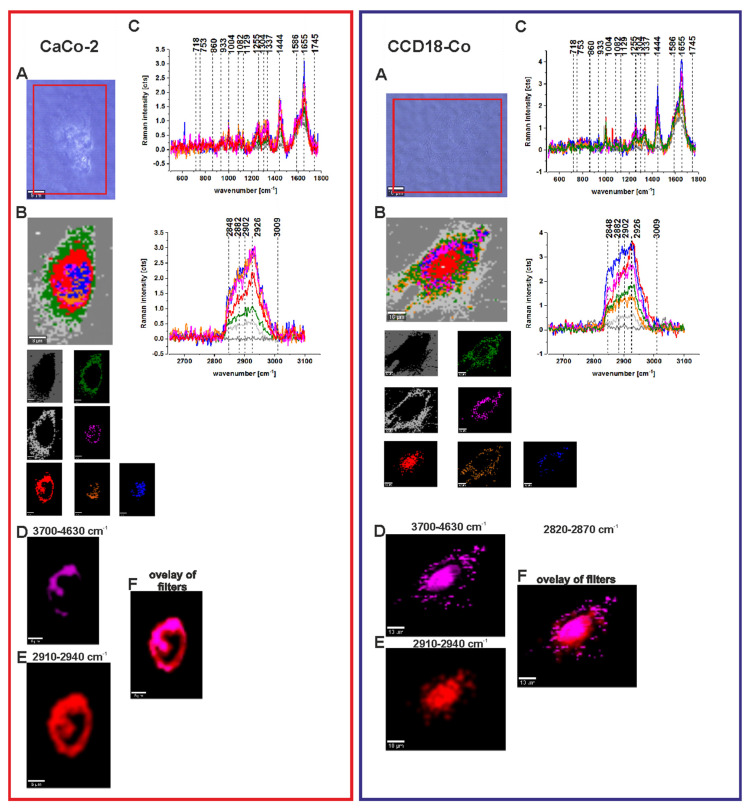
Microscopy image (**A**), Raman image constructed based on CA method and Raman images of all clusters identified by CA, nucleus (red), mitochondria (magenta), lipid-rich regions (blue and orange), membrane (light gray), cytoplasm (green), cell environment (dark gray) (**B**), Raman spectra typical of all clusters for low- and high-frequency regions (**C**), Raman image of ZnPcS_4_ phthalocyanine distribution based on filter 3700–4630 cm^−1^ (**D**), Raman image of the nucleus based on filter 2910–2940 cm^−1^, (**E**) overlay of ZnPcS_4_ phthalocyanine, and nucleus distribution filters, (**F**) the concentration of ZnPcS_4_ in a medium c = 9 × 10^−6^ M.

To check if the ZnPcS_4_ concentration can affect the localization of the photosensitizer inside cells, we performed the same experiments for the higher concentration of ZnPcS_4_ (c = 9 × 10^−6^ M). Figure 12 shows the results obtained for human normal and cancerous colon cells CCD18-Co and CaCo-2, respectively.

Once again, one can see from Figure 12 that adding the photosensitizer does not disturb the vibrational features of normal and cancerous human colon cells (panel C), but the most important finding of this experiment is that, for the higher concentration of ZnPcS_4_, the localization of the photosensitizer (panel D) is different and the localization inside the nucleus is predominant (panels E and F). As for the lower concentration, this conclusion was true for both normal and cancerous human colon cells (CCD18-Co and CaCo-2 respectively). 

To summarize, for normal and cancerous human colon cells, we proved that the localization of ZnPcS_4_ is concentration-dependent and so different mechanisms of cell death should be taken into account.

In PDT, an appropriate Pcs should be capable of inducing efficient formation of ROS after irradiation. Because ROS formation is a local effect, due to their short life-time and minimal radius of action, knowledge about the localization of the photosensitizer is crucial to the understanding of the mechanisms of programming cell death. It has been shown that many factors affect the complexity of the localization process, such as the central atom, substituents present in the macrocycle, and cell type (including cancer type and stage) [65]. Moreover, it has been proven that cellular uptake is a time- and concentration-dependent process [66]. This is why all of the studies regarding the PC localization are so important for future PDT development. Our results represent, therefore, another voice contributing to this lively discussion.

In our experiments, we show that, for the low ZnPcS_4_ phthalocyanine concentration (c = 1 × 10^−6^ M), the localization of the photosensitizer applies to mitochondria and lipid structures. This finding is consistent with the results proving that mono- or polysubstituted ZnPcs are mainly localized in the mitochondria and/or lysosomes in the Golgi apparatus, and, at a lower concentration, in the endoplasmic reticulum (ER) [67,68,69,70,71,72,73,74].

The mitochondrial-targeted PDT using ZnPcs was investigated by Muli et al. and Lan et al. [70,71].

It is well known also that mitochondrial homeostasis is essential for cell viability. This is why the mechanisms involving PDT damage to this organelle including caspase activation, deregulation in the expression of Bcl-2 family proteins, and reduction of mitochondria membrane potential are so interesting and have been reported in the literature after ZnPcs PDT [72,75]. 

Although the role of ER as a Pcs location is not clear, we have to remember that the ER is a key organelle involved in the folding and trafficking of newly synthesized proteins as well as in the maintenance of Ca^2^+ homeostasis, which is why ER photodamage after PDT can contribute to apoptotic cell death. An increase in the intracellular Ca^2^+ concentration and higher expression levels of ER stress marker proteins were reported after PDT treatment by using ZnPcs [76].

For the higher ZnPcS4 phthalocyanine concentration, we proved the preferential localization in the nucleus, which is the most important organelle. These results are in agreement with the data obtained by Kuzyniak et al. [77] and Machado et al. [78].

The nucleus is the largest cellular organelle, storing genetic information in the form of DNA. Singlet oxygen generated during PDT treatment is able to react with DNA by inducing guanine-to-thymine transversions due to 8-oxo-7,8-dihydro-20-deoxyguanosine generation. Nucleotide excision repair, base excision repair, and mismatch repair are implicated in the correction of DNA lesions induced by ^1^O_2_. At the molecular level, ^1^O_2_ is also able to induce the expression of genes involved in the cellular response to oxidative stress, such as NF-kB (nuclear factor kappa-light-chain-enhancer of activated B cells), c-fos (proto-oncogene), and c-jun (protein encoded by the JUN gene), and genes involved in tissue damage and inflammation such as ICAM-1 and interleukins 1 and 6. DNA–PDT damage-controlled cell death can be observed, e.g., as a result of nuclear condensation and DNA fragmentation during apoptosis. The most-studied pathway involves the nuclear translocation of apoptosis-inducing factor [79,80,81]. 

### 3.6. Transient Absorption 

**Figure 13:**
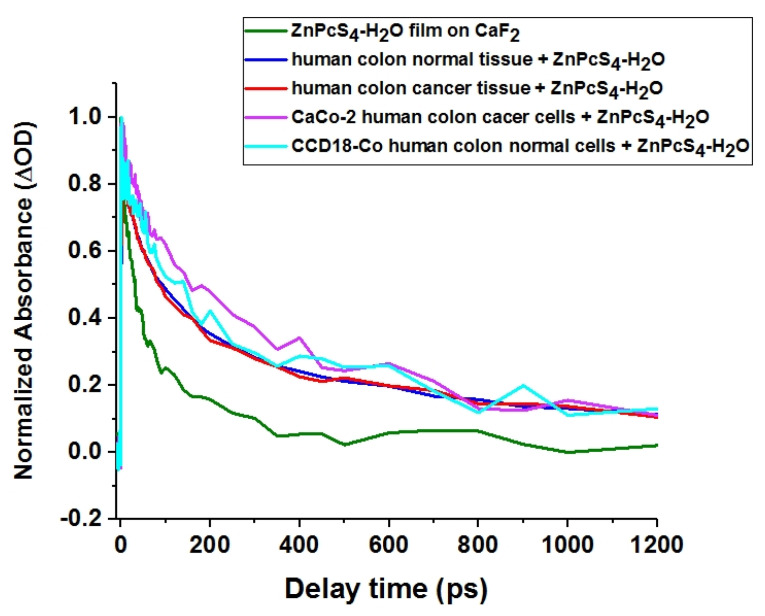
The transient absorption kinetic traces of ZnPcS_4_ film on CaF_2_ substrate (green line), CaCo-2 cells incubated with ZnPcS_4_ at c = 1 × 10^−4^ M (violet line), CCD18-Co cells incubated with ZnPcS_4_ at c = 1 × 10^−4^ M (turquoise line), normal colon tissue soaked with an aqueous ZnPcS_4_ solution at c = 1 × 10^−4^ M (blue line), cancerous colon tissue soaked with an aqueous solution at c = 1 × 10^−4^ M (red line). Pump and probe wavelength and energies were 650 nm (200 nJ) and 570 nm (15 nJ), respectively.

The impact of biological interfaces such as normal and cancerous colon tissues and cells on excited state lifetime of ZnPcS4 was investigated by means of transient absorption spectroscopy. Figure 13 shows the transient absorption kinetic traces of ZnPcS_4_ film on CaF_2_ substrate (green line), CaCo-2 cells incubated with ZnPcS_4_ at c = 1 × 10^−4^ M (violet line), CCD18-Co cells incubated with ZnPcS_4_ at c = 1 × 10^−4^ M (turquoise line), normal colon tissue soaked with an aqueous ZnPcS_4_ solution at c = 1 × 10^−4^ M (blue line), cancerous colon tissue soaked with an aqueous ZnPcS_4_ solution at c = 1 × 10^−4^ M (red line) for pump and probe wavelength 650 nm (200 nJ) and 570 nm (15 nJ), respectively.

ROS form as a result of reactions of the long-lived triplet state of the sensitizer with biomolecules in the presence of oxygen (type I reactions), or directly with oxygen in its ground triplet state (type II reactions). Therefore, the long excited-state lifetime of the photosensitizer is expected to favor photosensing efficiency. As the excited state lifetime depends on the surrounding of the chromophore, studying lifetimes in environments such as cells or tissues helps us to assess the potential effectiveness of the photosensitizer in vivo. For this reason, we measured the transient absorption of cancerous (CaCo-2) and normal (CCD18-Co) cells incubated with ZnPcS_4_, and cancerous and normal colon tissues soaked with an aqueous solution of ZnPcS_4_. We compared the kinetic traces of ZnPcS_4_ in biological environments with those of ZnPcS_4_ film on a bare CaF_2_ substrate (see Figure 13). The positive ΔA signals observed for all decay curves arise mainly from the excited state absorption because the probe at 570 nm covers only the edge of the ground state absorption spectrum of ZnPcS_4_ (see Figure 1). However, ground state bleaching also participates in the ΔA signal, reducing the absolute value of the ΔA. The decay of ΔA signal at 570 nm was three exponential and fitted with time constants varying from single ps to hundreds of ps. Table 2 shows the relaxation time constants for ZnPcS_4_ phthalocyanine obtained in our experiments.

One can see from Table 2 that the shorter time constant varies from 3.0 ps for 4.4 ps. Longer time constants of 424 and 432 ps were determined for cancerous cells and tissue, respectively. For cells and tissues, the determined lifetimes are 4.4 ± 3.1 ps, 65 ± 32 ps, and 424 ± 138 ps for CaCo-2 cancer cells; 3.0 ± 2.5 ps, 70 ± 32 ps, and 340 ± 89 ps for CCD18-Co normal cells; 3.6 ± 0.25 ps, 74.0 ± 9.0 ps, and 432.0 ± 98 ps for cancerous human colon tissue; and 3.0 ± 0.25 ps, 53.5 ± 5.7 ps, and 346.0 ± 38 ps for normal human colon tissue. The relative amplitudes of the determined time constants are also presented in Table 2. The longest time constant observed for samples of tissues and cells should be assigned to the excited single state (S_1_) lifetime of the photosensitizer. In contrast, for the aqueous solution, the long-time constant of few hundred ps is not observed due to fast S_1_→S_0_ internal conversion occurring in aggregates of ZnPcS_4_ [14,54]. While we are not able to measure the triplet state lifetime due to limited length of our delay stage, the increase S_1_ state lifetime should lead to higher yield of triplet state formation. The observed elongation of excited state lifetime is similar to that observed for ZnPcS_4_ monomers in micelles and in DMSO [14,54]. The most probable explanation of this behavior is disaggregation caused by interactions between ZnPcS_4_ molecules and biological interfaces. This mechanism is supported by the presence of observable fluorescence presumably having contribution from both ZnPcS_4_ monomers and non-cofacial aggregates (Figure 6C and Figure 7C). If this is the case, then the smaller amount of aggregates should facilitate interactions between the triplet state of photosensitizer and molecular oxygen as aggregates are more difficult to be penetrated by oxygen. The increase of S1 lifetime due to presence of ZnPcS4 monomers at biological interfaces should lead to rise of quantum yield of T1 because of less efficient S1→S0 internal conversion which is a competitive process for intersystem crossing S1→T1. The relative amplitude of the longest time constant assigned to the S1 lifetime varies from 38% to 65% for all ZnPcS4 at biological interfaces. This means that the value of triplet state quantum yield for ZnPcS4 at biological interfaces can be estimated to vary from 0.38 × 0.3 to 0.65 × 0.3 where 0.3 is reported value for ZnPcS4 in disaggregating solvent—DMF [82]. The value of triplet state quantum yield formation for ZnPcS4 at biological interfaces is expected to be much higher than that for pure aggregates in which very efficient internal conversion occurs. Apart from the a few hundred picosecond time constant assigned to S1 state of ZnPcS4 monomer, we observe two shorter time constants. The time constant in the range of 30–75 ps we assign to vibrational cooling. The time constants in the range of 3–5 ps may arise from internal conversion of aggregates. The observations presented for biological samples are in agreement with those made for human breast tissues by Abramczyk et al. [52,53,83]

To summarize, the presence of biological interfaces leads to an elongation of the excited state lifetime, which is beneficial for application in PDT, and the biological environment plays a crucial role in energy dissipation mechanisms in biological systems.

## 4. Conclusions

Raman imaging and spectroscopy were successfully used to characterize and differentiate normal and cancerous human colon tissues and cell lines based on vibrational features. Moreover, substructures of human colon single cells such as the nucleus, mitochondria lipid-rich regions, membrane, and cytoplasm can be precisely visualized based on the Raman spectra. Detailed biochemical information on human tissue samples can be obtained using Raman imaging for human colon tissues and cells without and after adding the ZnPcS_4_ photosensitizer. Fluorescence-based images accurately correspond to the Raman images, confirming the location of nucleus and lipid-rich structures. Based on Raman/fluorescence spectra and the images we obtained, we gleaned new information about the photosensitizer localization, concentration, and aggregation in normal and cancerous human colon tissues and cells. For normal human colon tissue samples, in contrast to cancerous ones, we observed lower affinity to ZnPcS_4_ phthalocyanine and the lower Raman/fluorescence signals typical of ZnPcS_4_. The higher concentration in cancerous tissue was concomitant with a blue-shift of the maximum peak position of the photosensitizer (689 nm for cancerous tissue and 691–695 nm for normal tissue; excitation wavelength: 532 nm). For both types of samples, the signal was observed in the monomer region, confirming the excellent properties of ZnPcS_4_ for PDTF. For colon cells, in experiments with a low concentration of ZnPcS_4_ photosensitizer (c = 1 × 10^−6^ M), the phthalocyanine was located in the mitochondria/lipid structures; for the higher concentration (c = 9 × 10^−6^ M), localization inside the nucleus was predominant. ZnPcS_4_ in the presence of biological interfaces is characterized by longer excited state lifetimes in comparison to aqueous solutions and bare ZnPcS_4_ films on CaF_2_ substrates. This finding is beneficial for the application of ZnPcS_4_ in PDT, and indicates that the biological environment plays a crucial role in energy dissipation mechanisms. 

## Figures and Tables

**Table 1:** The tentative assignments of Raman peaks differentiating the noncancerous and the cancerous tissues of human colon samples. Abbreviations: C—cancerous tissue, N—noncancerous tissue of human colon.

Wavenumber [cm^−1^]	Tentative Assignments	Type of Human Colon Tissue
749	nucleic acids, DNA, tryptophan, and nucleoproteins	C ↑
829/849	tyrosine	N ↑
858	stretching mode of the phosphate group	N ↑
872/938	stretching vibrations for the amino acids proline and valine and polysaccharides	N ↑
1004	phenylalanine	C ↑
1085	phosphodiester groups in nucleic acids	C ↑
1129	saturated fatty acids	C ↑
1260	Amide III (C–N stretch + N–H bend)	N ↑
1337	CH_3_CH_2_ wagging vibrations of collagen	C ↑
1445	lipids and proteins	N ↑
1557	Amide II (N–H bend + C–N stretch)	C ↑
1585	CN_2_ scissoring and NH_2_ rock of mitochondria and phosphorylated proteins	C ↑
1655	Amide I (C=O stretch)	N ↑
1745	C=O group of lipids and lipids esters.	N ↑
2848, 3009	C–H bonds of lipids, glycogen, proteins, RNA, and DNA.	N ↑
2931	Proteins	C ↑

**Table 2:** Relaxation time constants for ZnPcS4 phthalocyanine. The relative amplitudes for time constants are given in parentheses.

Sample	Time Constants
ZnPcS_4_ film on CaF_2_	3.97 ± 1.2 ps (28%), 32.3 ± 6.3 ps (44%), 190 ps ±46 (28%)
CaCo-2 cells incubated with ZnPcS_4_	4.4 ± 3.1 ps (7%), 65 ± 32 ps (28%), 424 ± 138 ps (65%)
CCD18-Co cells incubated with ZnPcS_4_	3.0 ± 2.5 ps (7%), 70 ± 32 ps (31%), 340 ± 89 ps (62%)
Cancerous colon tissue soaked with ZnPcS_4_ solution	3.6 ± 0.25 ps (25%), 74.0 ± 9.0 ps (37%), 432.0 ± 98 ps (38%)
Normal colon tissue soaked with ZnPcS_4_ solution	3.0 ± 0.25 ps (21%), 53.5 ± 5.7 ps (36%), 346.0 ± 38 ps (43%)
